# Outpatient Video Visits During the COVID-19 Pandemic: Cross-Sectional Survey Study of Patients’ Experiences and Characteristics

**DOI:** 10.2196/49058

**Published:** 2024-03-27

**Authors:** Stefanie C van den Bosch, Demi van Dalen, Marjan Meinders, Harry van Goor, Stefaan Bergé, Martijn Stommel, Sandra van Dulmen

**Affiliations:** 1 Department Oral and Maxillofacial Surgery Radboud University Medical Center Nijmegen Netherlands; 2 Department of Surgery Radboud University Medical Center Nijmegen Netherlands; 3 IQ Healthcare Radboud University Medical Center Nijmegen Netherlands; 4 Department of Primary and Community Care Radboud University Medical Center Nijmegen Netherlands; 5 Faculty of Caring Science, Work Life and Social Welfare University of Boras Boras Sweden; 6 Nivel Netherlands Institute for Health Services Research Utrecht Netherlands

**Keywords:** telemedicine, video visit, remote consultation, eHealth, patient-centered care, COVID-19, mobile phone

## Abstract

**Background:**

During the first lockdown of the COVID-19 pandemic, an exponential increase in video consultations replacing in-person outpatient visits was observed in hospitals. Insight into patients’ experiences with this type of consultation is helpful for a broad, sustainable, and patient-centered implementation of video consultation.

**Objective:**

This study aims to examine patients’ experiences with video consultation during the COVID-19 pandemic and identify discriminative patient and consultation characteristics to determine when video consultation is most feasible.

**Methods:**

A cross-sectional survey study was conducted. Patients aged ≥18 years and scheduled for a video consultation at the outpatient clinic of a Dutch university medical center from August 2020 to December 2020 for all medical specialties were eligible. Patients’ experiences were explored through a study-specific survey using descriptive quantitative statistics. Open-ended questions were qualitatively analyzed and thematically categorized into appreciated aspects and aspects for improvement. Discriminative patient and consultation characteristics were identified using 3 distinctive survey items. Characteristics of patients who scored and those who did not score all 3 items positively were analyzed using binary logistic regression.

**Results:**

A total of 1054 patients were included in the analysis. Most patients (964/1054, 91.46%) were satisfied with their video consultation, with a mean overall grade of 8.6 (SD 1.3) of 10. In the qualitative analyses, 70.02% (738/1054) of the patients cited aspects they appreciated and 44.97% (474/1054) mentioned aspects for improvement during their consultation. Patients with better self-rated health reported a positive evaluation significantly more often (*P=*.001), which also held true for other medical specialties (vs surgical and nonsurgical specialties; *P*<.001).

**Conclusions:**

Video consultation was perceived as highly satisfactory by patients during the COVID-19 pandemic, with the best experience reported by healthy participants and those undergoing their first consultation. Appreciated aspects are mainly at the individual professional level, organizational level, and innovation level itself. The aspects that were mentioned for improvement can be changed for the better.

## Introduction

### Background

In the Netherlands, the COVID-19 pandemic led to the first lockdown from March 2020 to June 2020 and the second lockdown from mid-December 2020 to April 2021. Throughout these periods, in-person visits were minimized to reduce the risk of potential virus transmission. As a substitute for in-person visits, the use of video visits significantly increased across many medical specialties.

Video visits were already in practice on a limited scale in a wide range of contexts: for speech evaluation in patients with cleft palate [1], genetic counseling [2], follow-up after facial plastic surgery [3], and postoperative wound assessment [4]. Video visits were found to be satisfactory for patients [5] and enabled empathetic patient-professional relationships remotely [1,6,7]. However, large-scale implementation in daily practice was found to be challenging owing to the multilevel complexity of implementation, where people, organizations, and technology continuously interconnect and develop [8-11]. For example, attitudes and beliefs of individual professionals have been shown to act as both facilitators and barriers in the implementation of eHealth applications [8]. Furthermore, video visits appeared to be particularly successful in follow-up appointments, when a preexisting relationship of trust is established between the patient and clinician [10]. For instance, video visits were more easily adopted in follow-up care after cancer surgery compared with a multidisciplinary context of antenatal diabetes care [10].

Owing to the pandemic-driven, accelerated application of video visits, many clinicians gained experience with this mode of health care delivery. This way of providing care offers several advantages, including saving travel time and costs for both patients and their companions and the efficient use of health care resources, such as outpatient clinic space and support [12-14]. As we move into the post–COVID-19 era, video visits are expected to persist as a routine practice, but large-scale use has seemed to stagnate, presumably owing to a lack of guidance, vision, and attention to patients’ needs, as observed in the United States [15,16]. The use and implementation of video visits are expected to be most successful when tailored to the needs of patients, clinicians, and health care organizations [17,18]. Therefore, it is crucial to understand patients’ perspectives and experiences with video visits and identify specific patient groups that show a greater or lesser degree of appreciation for and suitability to video visits [19]. Large studies with diverse patient populations covering all medical specialties need to be conducted to learn more about patients’ perspectives [16,18]. However, we are concerned that only a limited number of studies have been published that evaluated video visits for patients within large, diverse populations. Consequently, this study contributes significantly to the existing body of knowledge in this area [20-22].

### Aims

The primary aim of this study was to examine patients’ evaluations of video visits in the context of the COVID-19 pandemic. The secondary aim was to identify patient groups for whom video visits are relatively more suitable, given their positive experiences. With these results, the first step toward patient-tailored choices for type of visit can be made.

## Methods

### Study Design and Population

A cross-sectional study was conducted from August 2020 to December 2020 at the Radboud University Medical Center (Radboudumc). Zaurus was used as the video visit app, which is compatible with all smartphones and tablets. Patients were invited via email to register and download the app.

Patients scheduled for a video visit were automatically selected based on the registered mode of visit. Links to the questionnaire were sent by an independent research firm (Expoints) on behalf of the Radboudumc. Selected patients received the survey within 8 days after their visit via email to evaluate the visit and collect their sociodemographic details. The survey had to be completed within 2 weeks, and a reminder was sent 1 week after the initial invitation. An incomplete survey could be saved to be completed later (within 2 weeks). No reminder for completion was sent.

All patients aged ≥18 years who received a video visit at an outpatient clinic at Radboudumc were eligible for inclusion. When a patient had multiple video visits in the selected period, the most recent video visit was selected.

Patients were excluded if they had cognitive problems; had difficulties with reading and understanding Dutch owing to a hindering language barrier; were deceased at the time of selection; completed a survey regarding video visits in the 180 days before the start of our study; completed a survey regarding their admission experience or experience with an in-person visit 30 days before the start of our study; or were admitted to the hospital, as priority was given to the patient experience survey regarding admission. In addition, when the video visit was a follow-up visit after giving birth or when the video visit was replaced with another visit modality, the patient was excluded.

### Ethical Considerations

All patients participated voluntarily and anonymously in the survey and gave informed consent to use their data in accordance with the General Data Protection Regulation. Ethics approval was requested and waived by the local Medical Research Ethics Committee of Radboudumc (CMO [committee on research involving human subjects] Oost-Nederland; registration number 2021-8415).

### Survey

A combined survey was used, which consisted of the Patient Experience Monitor (PEM) for adult outpatient experience [23], developed by the Dutch Federation of University Medical Centers, and the patient satisfaction survey for video visits created by Hanna et al [7]. This combined survey was constructed after extensive deliberation by an expert panel. In this process, a literature review of surveys specifically about video visits was performed. The experts found the survey by Hanna et al [7] to be the most suitable for the aim of our study.

The PEM survey was constructed by adapting a validated Picker Institute survey following a comprehensive theory-driven approach of item selection by an expert panel, cognitive interviews with patients, analysis of psychometric properties, and member checking. This survey of 14 items represents eight key domains of person-centered care: (1) fast access to reliable health care advice; (2) effective treatment delivered by trusted professionals; (3) continuity of care and smooth transitions; (4) involvement and support for family and caregivers; (5) clear information, communication, and support for self-care; (6) involvement in making decisions and respect for preferences; (7) emotional support, empathy, and respect; and (8) attention to physical and environmental needs [24]. The Picker Institute surveys are measures for evaluating patients’ experiences in outpatient and inpatient clinical care and have been validated and extensively used in university medical centers in the Netherlands since 2019 [25]. The PEM survey was adapted to the videoconferencing setting by rephrasing the questions. Overall, 2 items were open-ended questions (Multimedia Appendix 1). Quantitative analyses of the PEM survey were based on individual survey items, whereas open-ended questions were analyzed using qualitative methods.

The 13-item survey by Hanna et al [7] is designed according to the principles of survey development for telemedicine to evaluate patients’ experiences with video visits in pain clinics [26]. This survey was translated into Dutch, and 1 item was removed, as it was already covered by the PEM survey (Multimedia Appendix 1, items 16-27). In total, 4 items were rephrased based on the advice of the patient communication experts. Analysis of the survey by Hanna et al [7] is based on an overall sum score, where a higher overall sum score represents greater satisfaction. For correct analysis and to calculate an overall sum score, the 3 negatively phrased questions (items 17, 21, and 23) were reversed (eg, “No, definitely not” was converted to “Yes, definitely”), according to protocol.

Finally, a question was added to assess the visit by assigning a score on a scale ranging from 1 to 10 (with 10 being most positive). The survey was conducted according to CHERRIES (Checklist for Reporting Results of Internet-Based e-Surveys; Multimedia Appendix 2) [27].

### Statistical Analysis

#### Quantitative Analysis

Descriptive statistics were used for the closed-ended items (Multimedia Appendix 1, items 1-11 and 16-27). To identify patient and visit characteristics associated with positive evaluation of video visits, 4 authors found consensus upon the 3 key items from the survey by Hanna et al [7] that stood out the most (item 18: “the care I received by a video visit was just as good as with an in-person appointment”; item 22: “I was comfortable talking by video to the healthcare professional”; item 27: “I would recommend the video visit option to other patients”). Throughout the paper, these 3 items have been referred to as “crucial” components of the survey by Hanna et al [7], as they best displayed a positive experience.

Patient and visit characteristics were determined for the group answering the 3 crucial items positively. In this analysis, the following characteristics were included: sex, age category, level of education, self-rated health, type of visit (first vs follow-up), and medical specialty (surgical, nonsurgical, or other). High self-rated health was defined as a score that indicates “very well” or “excellent.”

Statistical analysis was performed using SPSS Statistics (version 25; IBM Corp). Binary logistic regression analysis was used to calculate differences in patient and visit characteristics between the patient subgroup that scored positively on all crucial items and the patient subgroup that did not score positively, as the dependent variable was not normally distributed.

#### Qualitative Analysis

Qualitative analysis was performed on the open-ended questions (items 13 and 14) to identify appreciated aspects and aspects for improvement for video visits. Overall, 2 authors (SCvdB and DD) independently categorized all the answers into six categories of factors that influence the implementation of innovations: (1) the innovation itself, (2) the individual professional, (3) the patient, (4) social context, (5) organizational context, and (6) economic and political context [28]. In case of conflicts in the categorization, consensus was reached through discussion between the authors. Responses including multiple levels within a single response were counted as individual items. Examples of answers for both aspects in each category have been cited in the Results section.

## Results

### Quantitative Results

From August 2020 to December 2020, a total of 1244 surveys were completed, with a response rate of 28.32% (1244/4392). After excluding 15.27% (190/1244) of the patients who reported that the visit was either a telephone consultation or replaced by telephone after technical difficulties, 84.73% (1054/1244) of the surveys were used in the analysis.

Table 1 shows the patients’ demographics. An equal distribution across age categories was observed. Clinical genetics, neurology, and medical oncology accounted for 65.84% (694/1054) of the total number of evaluated video visits, whereas the distribution across the other medical specialties varied widely. The numbers of first and follow-up visits were equal, with most follow-up visits (480/1054, 45.54%) performed by a known clinician. After a video visit, 36.91% (389/1054) of the patients had to make an appointment for an additional in-person visit or medical examination. A follow-up visit via video was planned in 40.32% (425/1054) of the evaluated video visits. Clinicians from medical oncology and neurology more frequently scheduled an in-person follow-up visit for their patients—18.6% (40/215) and 23.3% (50/215), respectively. Moreover, in 39.7% (69/174) and 14.4% (25/174) of cases, the visits provided by clinical genetics and neurology respectively, were followed by a consecutive visit for additional (diagnostic) testing.

The overall grading for the video visit had a mean of 8.6 (SD 1.3; median 9) of 10. For 5 PEM items, >80% of the patients answered positively, that is, patients waited no longer than 5 minutes, clinicians had read their medical records well, patients received understandable answers, patients trusted the clinician, and patients had enough time to discuss their problems with the clinician. Refer to Table 2 for details about the responses of patients.

For the remaining 6 items, more than 21% stated that the item was either not applicable or answered positively. For instance, 80.25% (829/1033) indicated not receiving any new medication for the question about whether the professional explained the adverse effects of new medication. Analysis of the items in the survey by Hanna et al [7] showed that 91.46% (964/1054) of the patients was satisfied with their video visit, 66.98% (706/1054) found it to be just as good as an in-person visit, and 68.69% (724/1054) would recommend video visits to other patients, as shown in Table 3.

Of 1054 patients, 574 (54.46%) answered all 3 crucial items on the survey by Hanna et al [7] positively, 234 (22.2%) answered 2 of 3 positively, 138 (13.09%) answered only 1 item positively, 72 (6.83%) patients answered “not applicable” or responded negatively, and 36 (3.42%) responses were missing. Patient and visit characteristics of patients who positively answered all 3 crucial Hanna [7] items versus the group who did not are shown in Multimedia Appendix 3.

Results of the binary logistic regression are shown in Table 4. Negative association was found between the positive evaluation of a visit and the surgical and “other” medical specialties (B=−0.64; *P*<.001). Positive evaluation was also associated with the patient category who described their health as “very well” (B=1.12; *P*=.01). Sex, age, and educational status had no influence on whether a patient rated the visit positively. In addition, the reason for the visit was not found to have any influence on the positive evaluation of a video visit.

**Table 1:** Demographic characteristics.

Characteristics					Patients (n=1054), n (%)
**Sex**					
	Male			448 (42.5)	
	Female			606 (57.49)	
**Age (y)**					
	18-34			131 (12.43)	
	35-54			333 (31.59)	
	55-64			272 (25.81)	
	65-79			304 (28.84)	
	80-99			14 (1.33)	
**Educational level**					
	None			5 (0.47)	
	Primary			105 (9.96)	
	Secondary			378 (35.86)	
	Higher and university			468 (44.4)	
	Different			29 (2.75)	
	Missing			69 (6.55)	
**Self-rated health**					
	Excellent			95 (9.01)	
	Very well			167 (15.84)	
	Good			466 (44.21)	
	Not so well			255 (24.19)	
	Bad			49 (4.65)	
	Missing			22 (2.08)	
**Specialty**					
	**Nonsurgical medical specialisms**			498 (47.24)	
		Cardiology	33 (3.13)		
		Dermatology	3 (0.28)		
		Gastroenterology	16 (1.52)		
		Hematology	40 (3.79)		
		Internal medicine	66 (6.26)		
		Medical oncology	121 (11.48)		
		Nephrology	14 (1.33)		
		Neurology	153 (14.52)		
		Pulmonary medicine	25 (2.37)		
		Reproductive medicine	25 (2.37)		
		Rheumatology	2 (0.18)		
	**Surgical specialties**			101 (9.58)	
		ENT^a^	6 (0.57)		
		General surgery^b^	36 (3.41)		
		Gynecology	9 (0.85)		
		Neurosurgery	13 (1.23)		
		Obstetrics	1 (0.09)		
		OMF^c^ surgery	1 (0.09)		
		Orthopedic surgery	3 (0.28)		
		Plastic surgery	8 (0.76)		
		Urology	24 (2.27)		
	**Other medical specialties**			455 (43.17)	
		Clinical genetics	420 (39.85)		
		Psychiatry	29 (2.75)		
		Radiology	2 (0.18)		
		Radiotherapy	4 (0.38)		
**Type of visit**					
	First visit—referred by primary care			195 (18.5)	
	First visit—different health care professional			270 (25.62)	
	First visit—second opinion			15 (1.42)	
	Follow-up—known health care professional			480 (45.54)	
	Follow-up—new health care professional			71 (6.74)	
	Missing			23 (2.18)	
**Follow-up after video consultation**					
	Follow-up visit at the outpatient clinic			215 (20.39)	
	Follow-up video visit			425 (40.32)	
	Additional medical examination			174 (16.51)	
	Referred to the referring physician			41 (3.89)	
	There is no follow-up needed			126 (11.95)	
	I do not know			61 (5.79)	
	Missing			12 (1.14)	

^a^ENT: ear, nose, and throat.

^b^Includes trauma surgery, visceral surgery, surgical oncology, and vascular surgery.

^c^OMF: oral and maxillofacial surgery.

**Table 2:** Responses of patients to the Patient Experience Monitor (PEM) items^a^.

PEM item and response options			Responses (n=1054), n (%)
**Did you have to wait for the video visit?**			
	The professional was on time	719 (68.22)	
	I waited <5 min	143 (13.57)	
	I waited >5 min	62 (5.88)	
	I waited >10 min	42 (3.98)	
	I waited >15 min	63 (5.97)	
	I don’t know	22 (2.08)	
**Did the professional read your health record?**			
	Yes	991 (94.02)	
	Not thoroughly	25 (2.37)	
	No	2 (0.19)	
	I don’t know	21 (1.99)	
**Did you get answers you could understand?**			
	Yes	985 (93.45)	
	Most answers were understandable	26 (2.46)	
	No	2 (0.19)	
	No opportunity to ask questions	0 (0)	
	I didn’t have any questions	26 (2.47)	
**Did you trust the professional?**			
	Yes	1021 (96.87)	
	Less than I wanted	17 (1.61)	
	No	4 (0.38)	
**Was there enough time to talk to the professional about your disease or problem?**			
	Yes	1015 (96.29)	
	I needed more time	14 (1.33)	
	No	1 (0.09)	
	I don’t know	3 (0.28)	
**Could you participate in the decision making process?**			
	Yes	732 (69.45)	
	Less possible than I wanted	18 (1.71)	
	No	10 (0.95)	
	No need	272 (25.81)	
	I don’t know	7 (0.66)	
**Could a relative participate in the conversation?**			
	Yes	499 (47.34)	
	More possible than I wanted	8 (0.76)	
	Less possible than I wanted	4 (0.38)	
	No	8 (0.76)	
	There were no relatives present	241 (22.87)	
	No need	277 (26.28)	
	I don’t know	4 (0.38)	
**Did the professional explain the advantages and disadvantages of treatment or diagnostic process?**			
	Yes	743 (70.49)	
	Some aspects weren’t clearly explained	20 (1.89)	
	No	4 (0.38)	
	There was no explanation	2 (0.19)	
	No need	268 (25.43)	
**Did the professional explain the adverse effects of the new medication?**			
	Yes	170 (16.13)	
	There wasn’t enough explanation	23 (2.18)	
	No	11 (1.04)	
	I didn’t receive new medication	829 (78.65)	
**Did the professional tell you what to do after the visit?**			
	Yes	784 (74.38)	
	Not enough	14 (1.33)	
	No	7 (0.66)	
	No need	228 (21.63)	
	I don’t know	9 (0.85)	
**Could you speak to anyone of the hospital about your worries and anxieties?**			
	Yes	235 (22.29)	
	Sometimes	24 (2.28)	
	No	25 (2.37)	
	No need	758 (71.92)	

^a^The total in some sections is not 100% owing to missing responses.

**Table 3:** Responses of patients for the items on the survey by Hanna et al [7].

Items of the survey	Responses (n=1054), n (%)						
	Yes, definitely	Yes, I think so	Maybe yes, maybe no	I don’t think so	No, definitely not	Not applicable	Missing
I am satisfied with my video visit.	671 (63.66)	293 (27.79)	46 (4.36)	17 (1.61)	10 (0.95)	0 (0)	17 (1.61)
I worried about my privacy.	13 (1.23)	18 (1.71)	97 (9.20)	366 (34.72)	549 (52.09)	0 (0)	11 (1.04)
The care I received by video visit was just as good as with an in-person appointment.	321 (30.45)	385 (36.53)	162 (15.37)	102 (9.68)	23 (2.18)	44 (4.17)	17 (1.61)
It is important to me that the video visit saved me travel time.	428 (40.61)	280 (26.56)	191 (18.12)	107 (10.15)	32 (3.04)	0 (0)	16 (1.52)
It is important to me that the video visit saved me money.	169 (16.03)	179 (16.98)	276 (26.19)	140 (13.28)	106 (10.06)	165 (15.65)	19 (1.8)
I experienced technical problems during the video visit which caused a disturbance of the visit.	33 (3.13)	74 (7.02)	92 (8.73)	265 (25.14)	575 (54.55)	0 (0)	15 (1.42)
I was comfortable talking by video to the health care professional.	580 (55.03)	359 (34.06)	71 (6.74)	25 (2.37)	8 (0.76)	0 (0)	11 (1.04)
I would rather travel to have my next visit in-person than use video visit.	100 (9.48)	142 (13.47)	272 (25.81)	257 (24.38)	211 (20.02)	56 (5.31)	16 (1.52)
I was able to develop a friendly relationship with my health care professional.	305 (28.94)	477 (45.25)	209 (19.83)	43 (4.08)	4 (0.38)	0 (0)	16 (1.52)
I was able to explain my problems clearly to my healthcare professional during the video visit.	482 (45.73)	472 (44.78)	65 (6.17)	11 (1.04)	3 (0.28)	0 (0)	21 (1.99)
The video visit was convenient.	389 (36.91)	474 (44.97)	141 (13.38)	32 (3.04)	8 (0.76)	0 (0)	10 (0.95)
I would recommend the video visit option to other patients.	329 (31.21)	395 (37.47)	254 (24.09)	42 (3.98)	12 (1.14)	0 (0)	22 (2.09)

**Table 4:** Binary logistic regression model results^a^.

Variable		B (95% CI)	*P* value
Sex (male)		0.03 (0.78-1.37)	.83
**Age category (y)**			
	18-34	Reference	N/A^b^
	35-54	0.17 (0.76-1.86)	.45
	55-64	0.14 (0.72-1.85)	.55
	65-79	0.06 (0.65-1.73)	.81
	80-99	−0.17 (0.20-3.54)	.82
**Educational level**			
	None	Reference	N/A
	Primary	0.05 (0.16-7.07)	.96
	Secondary	0.05 (0.16-6.89)	.95
	Higher and university	−0.04 (0.15-6.22)	.96
	Different	−0.16 (0.11-6.43)	.88
**Self-rated health**			
	Excellent	Reference	N/A
	Very well	1.12 (1.39-6.72)	.01
	Good	0.52 (0.85-3.35)	.13
	Not so well	0.14 (0.61-2.15)	.66
	Bad	0.27 (0.69-2.50)	.41
**Specialty**			
	Nonsurgical	Reference	N/A
	Surgical	−0.64 (0.37-0.75)	<.001
	“Other”	−0.45 (0.38-1.06)	.08
**Type of visit**			
	First visit	Reference	N/A
	Follow-up visit	0.17 (0.86-1.65)	.30

^a^ Nagelkerke *R*^2^=0.08.

^b^N/A: not applicable.

### Qualitative Results

#### Overview

Table 5 displays the frequencies of appreciated aspects and aspects for improvement. Most patients (738/1054, 70.02%) cited appreciated aspects of the use of video visits in the open-ended questions. The most frequently cited appreciated aspects were expressed at the individual professional level, followed by the organizational context level and the innovation level. Few aspects were mentioned at the patient level, economic and political context level, and social context level. Approximately half of the patients (474/1054, 44.97%) cited aspects for improvement. Most were cited at the innovation, organizational context, and patient levels. In contrast, no improvable aspects were reported at the economic and political context level.

**Table 5:** Aspects that were appreciated and aspects for improvement obtained from patients’ experiences with video visits, extracted from answers to open-ended questions.

Level	Citations of aspects as appreciated^a^ (n=926)^b^, n (%)	Citations of aspects for improvement^c^ (n=388)^b^, n (%)
Innovation	185 (19.9)	138 (35.6)
Individual professional	399 (43.1)	46 (11.9)
Patient	82 (8.9)	91 (23.5)
Social context	11 (1.2)	17 (4.4)
Organizational context	204 (22)	96 (24.7)
Economic and political context	45 (4.9)	0 (0)

^a^Overall, 70.01% (738/1054) of the patients cited appreciated aspects.

^b^Responses including multiple levels within a single response were counted as individual items.

^c^Overall, 44.97% (474/1054) of the patients cited aspects for improvement.

#### Innovation

The perceived ease of use and audio-visual quality were frequently mentioned as appreciated aspects. The intuitive character of the app was seen as valuable, as not all patients were familiar with using web-based apps. Patients appreciated the audio-visual quality, allowing the video visit to be a good alternative for an in-person visit. However, not all the patients experienced the same ease of use, as the most reported aspect for improvement was poor audio and video quality, sometimes clearly caused by an unstable internet connection. Although Zaurus is compatible with all electronic devices, users have reported issues with video size specifically on smartphones (Textbox 1).

**Responses related to innovation.**

**Appreciated aspect**

“The application is straightforward and easy to understand. Conversation went well, the doctor even asked me if I could hear her well.” [Female; aged 18-34 y; clinical genetics]

**Aspect for improvement**

“The video connection was really bad. Almost immediately the app crashed, and the audio stuttered, so I could not understand what the doctor was saying. After two attempts, we continued the visit by telephone.” [Female; aged 65-79 y; excluded for further analysis; clinical genetics]

#### Individual Professional

Patients often mentioned what they valued in the clinician’s professional behavior, such as their attitude, and communicative style. In contrast, a lack of adequate or visible body language and lack of knowledge about someone’s medical history were mentioned as aspects for improvement (Textbox 2).

**Responses related to the individual professional.**

**Appreciated aspect**

“There is still a kind of personal touch in the contact, which is nice for the perception as well. The doctor radiated tranquility and was understanding, and she had read my personal record well. That gives me confidence.” [Female; aged 55-64 y; clinical genetics]

**Aspect for improvement**

“The doctor did not look at us during the video visit. Both my daughter, who was also present, and I had noticed. That felt a little awkward. He was mainly looking down (I guess at a file or something like that, which was in front of him).” [Female; aged 80-99 y; clinical genetics]

#### Patient

The possibility to have face-to-face interactions remotely was often mentioned as valuable, as patients were able to watch the clinicians’ nonverbal reactions. It made video visits a safe alternative for patients with a weak immune system, for example, during the pandemic. Personal lack of experience with video visits was a hindering factor, as not all patients were familiar with the use of videoconferencing apps. Some of them preferred an in-person visit, as they felt uncomfortable owing to inexperience (Textbox 3).

**Responses related to the patient.**

**Appreciated aspect**

“It is nice to see the doctor, but for a first meeting, it is something I need to get used to. However, this feels safer regarding the coronavirus and a vulnerable immune status.” [Female; aged 35-54 y; neurology]

**Aspect for improvement**

“I’d rather have the first visit in person. Maybe I’m old fashioned, but I prefer physical contact, even during this COVID pandemic. Feelings and emotions might be more difficult to pick up on screen.” [Male; aged 55-64 y; neurology]

#### Social Context

The possibility of the involvement of others, such as next of kin or other family members, was one of the mentioned appreciated aspects. Creating a culture in which a patient can share their preference or opt for a certain visit modality could stimulate the use of video visits. Patients expressed that they would like to have a say in choosing which visit modality they like, especially when the nature of the visit is sensitive (Textbox 4).

**Responses related to the social context.**

**Appreciated aspect**

“On time, pleasant conversation, space for questions, clear explanation. It was nice that my partner could join with his phone.” [Female; aged 18-34 y; reproductive medicine]

**Aspect for improvement**

“It was a shame they communicated the results by a video visit. I was shocked and found they acted a bit indignant about my reaction. I was not capable anymore to follow the conversation.” [Female; aged 18-34 y; clinical genetics]

#### Organizational Context

Internet-based assistance, clear instructions, and time management by the clinician during the visit were often mentioned as appreciated aspects at this level. Many patients were called in advance of the visit to check for technical problems. However, patients were not always informed correctly if the visit would start later than scheduled, and in some cases, patients received the link for the video visit just before the visit started, which was an aspect for improvement (Textbox 5).

**Responses related to the organizational context.**

**Appreciated aspect**

“The support was really good, as I am not so technical and there was enough explanation. Great.” [Female; aged 55-64 y; clinical genetics]

**Aspect for improvement**

“I would like to receive a notification when the doctor is held up, especially when it’s a first visit. Also, I would like to receive a heads up when I get another doctor than the one the appointment was originally scheduled with.” [Male; aged 55-64 y; medical oncology]

#### Economic and Political Context

Time and financial savings were identified as valuable aspects at the economic and political context level. Patients often cited less travel time and costs as beneficial. There were no improvable aspects reported at the economic and political context level (Textbox 6).

**Responses related to the economic and political context.**

**Appreciated aspect**

“It is pleasant that there is no need for traveling to the hospital (regarding travel time and travel distance) and still have ‘personal’ contact with the doctor through a video connection.” [Male; aged 35-54 y; neurology]

## Discussion

### Principal Findings

In this study, we comprehensively analyzed evaluations of visits via video to a tertiary clinic made by a large, diverse patient population, including appreciated aspects and aspects for improvement. Most patients (964/1054, 91.46%) evaluated the video visits positively, with significantly more positive evaluations when the visits were provided by a clinician from “other” medical specialties, as compared to surgical and nonsurgical specialties, or when the patient rated their health status as “very well.” The appreciated aspects were mostly at the individual professional level, whereas aspects for improvement were reported at the innovation level itself.

### Comparison With Previous Studies

Our finding that high self-rated health of patients is an influencing patient characteristic for suitability of video visits echoes the finding that patients with less complex, more straightforward clinical needs are more suitable candidates for video visits than those with complex, high-risk diseases [10,29]. Similar findings were identified in an oncological study wherein telemedicine was received favorably for low-acuity cancer care [16]. In contrast to findings that video visits appear to be more appropriate when the clinician knows the patient beforehand and when it is a follow-up visit [10], we found that the reason for the visit did not have any influence on whether patients rated the video visit positively. Remarkably, the medical specialty providing the visit was found to be a significant associated factor. Nonsurgical visits were found to be most suitable for telemedicine. One can imagine that these visits are less dependent on physical examination, such as internal medicine or dermatology, for instance, as these specialties can easily review laboratory abnormalities or skin disorders on screen [30]. In addition to specific aspects of the visit that may depend on the medical specialty, other dimensions or elements during the visit could affect the patient experience, such as the communication strategy used by the clinician [31]. Nonetheless, more studies are needed to get a clear overview about whether medical specialty is a truly discriminative characteristic or whether it is more dependent on the attitude of certain clinicians and patient groups.

Qualitative analysis of the open-ended questions revealed both facilitating and hindering factors for broad implementation and upscaling of video visits. Following Grol and Wensing [28], these factors were categorized into innovation, professional, patient, social context, organizational context, and economic and political context levels. Appreciation was mostly centered on the professionals’ skill in adapting communication to the video setup; however, there was scope for improvement among some individuals, as they might benefit from investing additional effort in making visual contact. The way in which the video visits were organized was also highly valued, especially for the provision of technical support to patients as and when needed.

The attitude of the clinician during the video visit was one of the most frequently mentioned aspects for improvement. The bedside manner, which may be better described as the “webside” manner, of a clinician refers to how the clinician behaves, approaches the patient, and communicates during the visit. Clinicians sometimes seem to lack awareness of how their nonverbal behavior looks on screen, as was also shown by a study that analyzed a large data set of >5000 patients [32]. Patients prefer increased expression of nonverbal empathy from clinicians when they show signs of distress. Inadequate nonverbal communication and body language are often reported as barriers for telemedicine adoption [3,33,34]. Despite expert recommendations dating back several years to raise awareness for nonverbal and paraverbal communication, our study also indicates that there is still considerable scope for improvement and training at the clinician level [31,35].

In accordance with several survey studies conducted during the pandemic, our response rate was low. This might be explained by the questionnaire fatigue that was frequently observed during the COVID-19 pandemic, as patients received multiple questionnaires and messages from the outpatient clinic, apart from research [36].

Regarding future perspectives, clinicians should seek guidelines to assess the suitability of a video visit, and the following recommendations might be useful. The Dutch Center of Expertise on Health Disparities recommends checking the patients’ digital skills beforehand, providing digital support, and evaluating whether the information is correctly understood through techniques such as “teach back” at the end of the visit [37]. Video consulting guidelines advise considering several factors while deciding whether video visits may be suitable, such as whether there is an established relationship with the patient, whether it entails nonurgent care, whether there is a need for physical examination, and whether there are factors in favor of the patient staying at home [38].

### Limitations

The findings of this study must be considered in the light of some limitations. First, our study might have been exposed to selection and sampling biases for several reasons. It was an “open” survey, where patients could decide voluntarily whether they would participate in the survey, which might have led to a sample of patients that is not representative of the entire population of the hospital. In addition, owing to the exclusion criteria, not all video visits were evaluated.

Second, the validity of the combined survey was not tested. As the analysis of our data was reported at the item level, calculating the internal consistency using Cronbach α was not applicable. PEM is known to be a validated survey; however, the psychometric properties of the survey by Hanna et al [7] are not known and should be determined. As the PEM items were rephrased to the videoconferencing setting, reliability of this new PEM survey will have to be reassessed.

The educational status of patients attending a university medical center is, in general, often higher than the mean educational status of the general population. In our study population, 44.4% (468/1054) of the patients were highly educated, compared with 30% in the Dutch population in 2018 [39]. As teaching hospitals and referring hospitals might serve different populations, the generalizability of our results might be limited, and further studies including different types of hospitals are recommended.

A total of 139 patients reported a failed video visit and noted that the visit was replaced by telephone. However, the exact number of times this occurred is not known, as not all patients might have reported this failure, which also may have resulted in selection bias.

In this evaluation, we deliberately focused on the patient evaluation of video visits. As it is known that patients and clinicians have different views about quality of information and visits [40-42], the clinicians’ point of view should also be explored for a comprehensive evaluation of the use of video visits. Health care providers, such as clinicians, might experience different barriers and facilitators compared with patients, thus influencing the successful implementation of video visits. Key barriers to successful implementation such as the lack of training and motivation to offer video visits need to be addressed [3,8,43,44].

### Conclusions

Video visits were perceived as highly satisfactory by patients during the COVID-19 pandemic, with the best experiences reported by healthy participants and participants who scheduled a visit with a clinician outside the realms of surgical and nonsurgical medical specialties, such as clinical genetics or radiotherapy. Appreciated aspects were mainly at the individual professional level, organizational level, and at the level of the innovation itself. The mentioned aspects for improvement can be changed for the better.

The findings cannot be directly generalized as they were collected in a university medical center with a specific patient population, but they provide additional results for understanding the suitability of video visits in a broad patient population. To be able to truly tailor the use of video visits to patients’ needs, a patient-centered perspective involving both patients and health care professionals is needed.

## Appendix

Multimedia Appendix 1 Survey on video visits during the COVID-19 outbreak (August 2020 to December 2020).

Multimedia Appendix 2 CHERRIES (Checklist for Reporting Results of Internet-Based e-Surveys) checklist.

Multimedia Appendix 3 Patient and visit characteristics of those who positively answered all 3 crucial items in the checklist by Hanna et al [7] versus the group that did not answer positively.

## Abbreviations

CHERRIES: Checklist for Reporting Results of Internet-Based e-Surveys
PEM: Patient Experience Monitor

## Data Availability

The data used in this study will be made available by the authors upon reasonable request.
