# Supplementary material for: Outpatient Video Visits During the COVID-19 Pandemic: Cross-Sectional Survey Study of Patients’ Experiences and Characteristics
Source: J Med Internet Res. 2024 Mar 27;26:e49058. doi: 10.2196/49058 (PMC10977342; doi:10.2196/49058)
Supplement: Multimedia Appendix 1 [file jmir_v26i1e49058_app1.docx]

**Multimedia Appendix 1. Survey video visit during the COVID-19 outbreak (August 2020 to December 2020).**

*1.Did you have to wait for the video visit, or was the healthcare professional on time?*

The healthcare professional was on time

I waited less than 5 minutes

I waited more than 5 minutes

I waited more than 10 minutes

I waited more than 15 minutes

I don’t know how long I had to wait

*2. Did the healthcare professional read your health record in preparation to visit?*

Yes, my record was well

My record was read, but not thoroughly

No, my record wasn’t read

I don’t know (anymore)

*3. Should you have questions for the healthcare professional, did you get answers you could understand?*

Yes, I could understand the answers

Most of the time the answers were understandable, sometimes they weren’t

No, the answers were not understandable

I had some questions for the healthcare professional, but I didn’t have the opportunity to ask those

I didn’t have any questions

*4. Did you trust the healthcare professional?*

Yes, I trusted the healthcare professional

I trusted the healthcare professional less than I wanted

No, I didn’t trust the healthcare professional

*5. Was there enough time to talk to the healthcare professional about your disease or problem?*

Yes, there was enough time

There was time, but I needed more time

No, there wasn’t time

I don’t know (anymore)

*6. Could you participate in the decision-making process about your treatment or diagnostic process?*

Yes, I could as much as I wanted

That was less possible than I wanted

No, I couldn’t

That was not necessary

I don’t know anymore

*7. Could a relative participate in the conversation about your treatment or diagnostic process?*

Yes, that was possible as much as I wanted

That was more possible than I wanted

That was less possible than I wanted

No, they couldn’t participate

There were no relatives present during the visit

That was not necessary

I don’t know anymore

*8. Did the healthcare professional explain the advantages and disadvantages of treatment or diagnostic process?*

Yes, this was clearly explained

Some aspects were not clearly explained

No, this was not explained

There was no explanation

That was not necessary

*9. Did the healthcare professional explain the adverse effects of the new medication?*

Yes, this was explained

There was some explanation, but not enough

No, there was no explanation

I did not receive new medication

*10. Did the healthcare professional tell you what to do after the visit? Did you had to make a new appointment or see your general practitioner for instance?*

Yes, we did speak about this

We did speak about this, but not enough

No, I would have preferred to speak about this

That was not necessary

I don’t know anymore

*11. Could you speak to anyone of the hospital about your worries and anxieties?*

Yes, I could talk to someone

Sometimes I could talk to someone, sometimes I couldn’t

No, I couldn’t talk to anyone

That was not necessary

*12. With which mark you would rate your video visit?*

1 means 'Very bad', 10 means 'Very good'.

1 2 3 4 5 6 7 8 9 10

*13. What did we do very well during your video visit? (not mandatory)*

*Please, do not mention any personal information such as names and telephone numbers.*

------------------------------------------------------------------------------------------------------------------------------------------------------------------------------------------------------------------------------------------------------------------------------------------------------------------------------------------------

*14. What could we improve during a video visit? (not mandatory)*

*Please, do not mention any personal information such as names and telephone numbers.*

------------------------------------------------------------------------------------------------------------------------------------------------------------------------------------------------------------------------------------------------------------------------------------------------------------------------------------------------

*15. What kind of visit did your video consult concern?*

First visit on referral of general practitioner or dentist

First visit on referral of another medical specialist

First visit, it concerned a second opinion on my own request

Follow-up visit with a previous known healthcare professional

Follow-up visit with a new healthcare professional

*16. I am satisfied with my video visit*

No, definitely not

I don’t think so

Maybe yes, maybe no

Yes, I think so

Yes, definitely

*17. I worried about my privacy*

No, definitely not

I don’t think so

Maybe yes, maybe no

Yes, I think so

Yes, definitely

*18. The care I received by video visit* *was just as good as with an in-person appointment.*

No, definitely not

I don’t think so

Maybe yes, maybe no

Yes, I think so

Yes, definitely

*19. It is important to me that the video visit saved me travel time.*

No, definitely not

I don’t think so

Maybe yes, maybe no

Yes, I think so

Yes, definitely

*20. It is important to me that the video visit saved me money.*

No, definitely not

I don’t think so

Maybe yes, maybe no

Yes, I think so

Yes, definitely

*21. I experienced technical problems during the video visit which caused a disturbance of the visit.*

No, definitely not

I don’t think so

Maybe yes, maybe no

Yes, I think so

Yes, definitely

*22. I was comfortable talking by video to the healthcare professional.*

No, definitely not

I don’t think so

Maybe yes, maybe no

Yes, I think so

Yes, definitely

*23. I would rather travel to have my next visit in-person than use video visit.*

No, definitely not

I don’t think so

Maybe yes, maybe no

Yes, I think so

Yes, definitely

Not applicable

*24. I was able to develop a friendly relationship with my healthcare professional.*

No, definitely not

I don’t think so

Maybe yes, maybe no

Yes, I think so

Yes, definitely

*25. I was able to explain my problems clearly to my healthcare professional during the video visit.*

No, definitely not

I don’t think so

Maybe yes, maybe no

Yes, I think so

Yes, definitely

*26. The video visit was convenient.*

No, definitely not

I don’t think so

Maybe yes, maybe no

Yes, I think so

Yes, definitely

27. *I would recommend the video visit option to other patients.*

No, definitely not

I don’t think so

Maybe yes, maybe no

Yes, I think so

Yes, definitely

*28. What did you agree with your healthcare professional about the follow-up after this visit?*

An appointment for follow-up visit in the out-patient clinic is made

An appointment for follow-up video visit is made

An appointment is made for additional medical examination

I will be referred back to the referral (e.g. general practitioner)

There is no follow-up needed

I don’t know what the follow-up will be

*29. What education did you take last (with diploma).*

Primary education

Secondary education

Higher education/university

Different

I did not take any education

*30. How would you rate your general health?*

My general health is excellent

My general health is very well

My general health is well

My general health is not so well

My general health is poor
