# Supplementary material for: Outpatient Video Visits During the COVID-19 Pandemic: Cross-Sectional Survey Study of Patients’ Experiences and Characteristics
Source: J Med Internet Res. 2024 Mar 27;26:e49058. doi: 10.2196/49058 (PMC10977342; doi:10.2196/49058)
Supplement: Multimedia Appendix 2 [file jmir_v26i1e49058_app2.docx]

**Multimedia Appendix 2.**

CHERRIES (Checklist for Reporting Results of Internet-Based e-Surveys) checklist.

| ***Item Category*** | ***Checklist Item*** | ***Explanation*** |
| --- | --- | --- |
| **Design** |  |  |
|  | Describe survey design | The study is a convenience sample; the questionnaire was distributed to all patients, aged 18 years and older, who received a video visit at an outpatient clinic at the Radboudumc. |
| **IRB (Institutional Review Board) approval and informed consent process** | IRB approval | Ethical approval was re-quested and waived by the local Medical Research Ethics Committee of the Radboudumc (CMO Oost-Nederland, registration number: 2021-8415). |
|  | Informed consent | Patients were informed of the purpose of the survey, anonymity, confidentiality and voluntarily filled out the questionnaire. |
|  | Data protection | The questionnaire was anonymous, and patients voluntarily filled out their personal information (such as sex and age). The researchers received an excel file with a randomized patient number, age and sex. The data file was stored on the secured drive of the department. Only specific research members of the team, who signed a confidentiality agreement, were able to view and analyze the anonymized data file. |
| **Development and pre-testing** | Development and testing | The questionnaires were automatically dispatched via email through the electronic patient record system |
| **Recruitment process and description of the sample having access to the questionnaire** | Open survey versus closed survey | Open survey. Patients can answer the questionnaire voluntarily by clicking on the link to the online questionnaire. |
|  | Contact mode | Patients were contacted via email to participate in the questionnaires, which were sent on behalf of Radboud by Expoints, an independent research firm. |
|  | Advertising the survey | The questionnaire is promoted through the official website of the Radboud University Medical Center. |
| **Survey administration** | Web/E-mail | Patients were contacted via email to participate in the questionnaires, which were sent on behalf of Radboud by Expoints, an independent research firm. |
|  | Context | The independent research firm Expoints sent emails on behalf of the Radboud University Medical Center. We only received the anonymized data file. |
|  | Mandatory/voluntary | Voluntary |
|  | Incentives | No incentives offered. |
|  | Time/Date | Data was collected from August 2020 up to December 2020. |
|  | Randomization of items or questionnaires | Question order was not randomized. |
|  | Adaptive questioning | N/A |
|  | Number of Items | 30 items |
|  | Number of screens (pages) | N/A |
|  | Completeness check | Not applicable option was offered. There was no completeness check before submission; therefore some items are missing. |
|  | Review step | Patients were able to go ‘back’ in the survey. |
| **Response rates** |  |  |
|  | Unique site visitor | N/A |
|  | View rate (Ratio of unique survey visitors/unique site visitors) | The survey is voluntary. The system cannot record the number of unique visitors, so the view rate cannot be calculated. Assumed is that the view rate is very low, since only patients eligible for filling out the questionnaire, were approached. |
|  | Participation rate (Ratio of unique visitors who agreed to participate/unique first survey page visitors) | Participation rate cannot be calculated. |
|  | Completion rate (Ratio of users who finished the survey/users who agreed to participate) | Completion rate cannot be calculated. Participants submit the questionnaire on behalf of their consent to participate. |
| **Preventing multiple entries from the same individual** | Cookies used | There were no cookies used. |
|  | IP check | The questionnaire was only send ones. If patients had multiple visits that could be reviewed by this questionnaire, only an invitation for the most recent visit was send. There is no IP check done. |
|  | Log file analysis | N/A |
|  | Registration | N/A |
| **Analysis** | Handling of incomplete questionnaires | Missing data was reported. |
|  | Questionnaires submitted with an atypical timestamp | We have a set deadline for data collection, the data filled in after this deadline was not included in the analysis. |
|  | Statistical correction | The dependent variable in this study was non-normal distributed and therefor binary logistic regression was used. |
