# Supplementary material for: Outpatient Video Visits During the COVID-19 Pandemic: Cross-Sectional Survey Study of Patients’ Experiences and Characteristics
Source: J Med Internet Res. 2024 Mar 27;26:e49058. doi: 10.2196/49058 (PMC10977342; doi:10.2196/49058)
Supplement: Multimedia Appendix 3 [file jmir_v26i1e49058_app3.docx]

**Multimedia Appendix 3.**

**Patient and visit characteristics of those who positively answered all 3 crucial items in the checklist by Hanna et al [7] versus the group that did not answer positively.**

|  |  | **all 3 crucial Hanna items positively answered**  **(n= 624)** | **not all 3 crucial Hanna items positively answered**  **(n= 430)** |  |
| --- | --- | --- | --- | --- |
|  |  |  |  |  |
| **Patient characteristics** | | **n (%)** | **n (%)** |  |
|  | |  |  |  |
| *Sex* | *Female* | 336 (58.5%) | 250 (56.3%) |  |
|  | *Male* | 238 (41.5%) | 194 (43.7%) |  |
| *Age (years)* | *18-34* | 71 (12.4%) | 59 (13.3%) |  |
|  | *35-54* | 194 (33.8%) | 134 (30.2%) |  |
|  | *55-64* | 146 (25.4%) | 116 (26.1%) |  |
|  | *65-79* | 155 (27%) | 131 (29.5%) |  |
|  | *80-99* | 8 (1.4%) | 4 (0.9%) |  |
| *Level of education* | *None* | 2 (0.3%) | 2 (0.5%) |  |
|  | *Primary* | 59 (10.3%) | 43 (9.7%) |  |
|  | *Secondary* | 213 (37.1%) | 159 (35.8%) |  |
|  | *Higher/university* | 253 (44.1%) | 201 (45.3%) |  |
|  | *Other* | 13 (2.3%) | 13 (2.9%) |  |
| *Self-rated health* | *Excellent* | 65 (11.3%) | 29 (6.5%) |  |
|  | *Very well* | 103 (17.9%) | 61 (13.7%) |  |
|  | *Good* | 246 (42.9%) | 208 (46.8%)^a^ |  |
|  | *Not so well* | 132 (23%) | 115 (25.9%) |  |
|  | *Bad* | 20 (3.5%) | 25 (5.6%) |  |
|  | |  |  |  |
| ***Visit*** ***characteristics*** | |  |  |  |
|  | |  |  |  |
| *Type of visit* | *First* | 292 (50.9%) | 176 (39.6%) |  |
|  | *Follow-up* | 269 (46.9%) | 264 (59.5%) |  |
| *Visited medical*  *specialism* | *Surgical* | 49 (8.5%) | 45 (10.1%) |  |
|  | *Non-surgical* | 234 (40.8%) | 245 (55.2%) |  |
|  | *Others* | 291 (50.7%) | 154 (34.7%) |  |

; ^a^ median value
